# Robust three-dimensional type-II Dirac semimetal state in SrAgBi

**DOI:** 10.1038/s41535-023-00549-8

**Published:** 2023-05-06

**Authors:** Zhixiang Hu, Junze Deng, Hang Li, Michael O. Ogunbunmi, Xiao Tong, Qi Wang, David Graf, Wojciech Radoslaw Pudełko, Yu Liu, Hechang Lei, Svilen Bobev, Milan Radovic, Zhijun Wang, Cedomir Petrovic

**Affiliations:** 1 Condensed Matter Physics and Materials Science Department, Brookhaven National Laboratory, Upton, NY 11973 USA; 2 Department of Materials Science and Chemical Engineering, Stony Brook University, Stony Brook, NY 11790 USA; 3 Beijing National Laboratory for Condensed Matter Physics and Institute of Physics, Chinese Academy of Sciences, Beijing, 100190 China; 4 University of Chinese Academy of Sciences, Beijing, 100049 China; 5 Swiss Light Source, Paul Scherrer Institut, CH-5232 Villigen, Switzerland; 6 Department of Chemistry and Biochemistry, University of Delaware, Newark, DE 19716 USA; 7 Center of Functional Nanomaterials, Brookhaven National Laboratory, Upton, NY 11973 USA; 8 Department of Physics and Beijing Key Laboratory of Opto-electronic Functional Materials & Micro-nano Devices, Renmin University of China, Beijing, 100872 China; 9 National High Magnetic Field Laboratory, Florida State University, Tallahassee, FL 32306-4005 USA; 10 Physik-Institut, Universität Zürich, Winterthurerstrasse 190, CH-8057 Zürich, Switzerland; 11 Present Address: School of Physical Science and Technology, Shanghai Tech University, Shanghai, China; 12 Present Address: Los Alamos National Laboratory, Los Alamos, NM 87545 USA

**Keywords:** Topological insulators, Electronic properties and materials

## Abstract

Topological semimetals such as Dirac, Weyl or nodal line semimetals are widely studied for their peculiar properties including high Fermi velocities, small effective masses and high magnetoresistance. When the Dirac cone is tilted, exotic phenomena could emerge whereas materials hosting such states are promising for photonics and plasmonics applications. Here we present evidence that SrAgBi is a spin-orbit coupling-induced type-II three-dimensional Dirac semimetal featuring tilted Dirac cone at the Fermi energy. Near charge compensation and Fermi surface characteristics are not much perturbed by 7% of vacancy defects on the Ag atomic site, suggesting that SrAgBi could be a material of interest for observation of robust optical and spintronic topological quantum phenomena.

## Introduction

Weyl semimetals (WSMs) and Dirac semimetals (DSMs) are of high current interest and are found at the critical point between topological and trivial insulators, featuring linear dispersion around touching points between conduction and valence bands; the Weyl points come in pairs and WSM requires breaking of either the time-reversal symmetry or the lattice inversion symmetry^1,2^. The touching points are protected by crystalline symmetries whereas other electronic states are removed by the spin-orbit coupling (SOC) which gaps the overlaps of the valence and conduction bands via removing multiple irreducible representations^3–5^. Emergent Dirac cones host highly mobile carriers with large Fermi velocities, exotic electronic states and are of interest for various applications such as optoelectronics or spin-charge conversion^6–13^. Strong SOC interaction is commonly found in heavy atomic species and partially scales with the atomic number *Z* as *Z*^4^^14,15^.

**Fig. 1:**
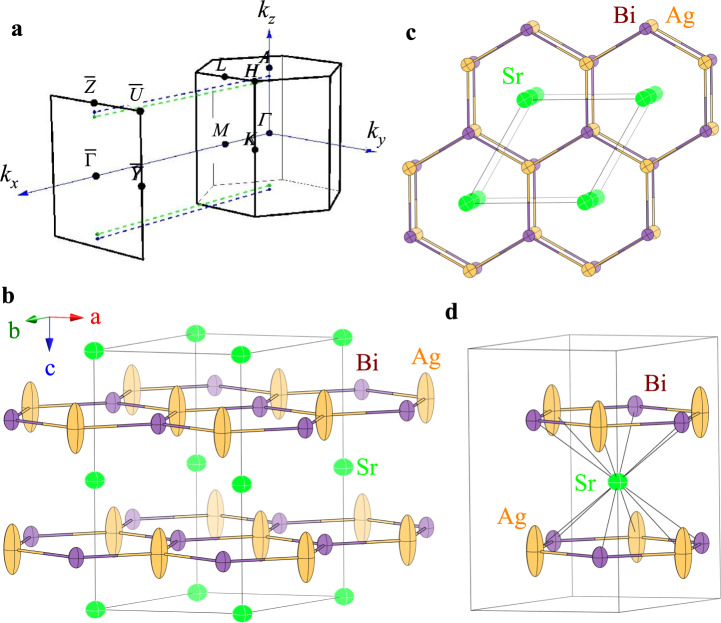
Reciprocal space and crystallographic unit cell of SrAgBi. **a** Reciprocal space of SrAgBi with projected surface Brillouin zone of (100) plane (Ref.^20^); type-II (type-IV) Dirac points are in close proximity and are shown by green (blue) dots. Hexagonal unit cell **b** of SrAgBi with inserted Ag-Bi rings between Sr layers and thermal ellipsoids from the single crystal X-ray refinement (see text). The unit cell can be viewed as stacks of Sr honeycombs along the hexagonal *c*-axis which maintain bulk inversion symmetries **c**; Ag-Bi hexagonal rings are inserted between them. Local environment of Ag atoms **d**.

SrAgBi crystallizes in the hexagonal crystal lattice with space group *P*6_3_/*mmc* (194)^16^. First-principle calculations predict a three-dimensional (3D) Dirac point about 0.1 eV above the Fermi level in the Γ-*A* direction whereas states at the Fermi level are dominated by the electron pocket at *M*^17^. The 3D Dirac semimetal state in SrAgBi is derived from a charge-balanced semiconductor Sr^2+^Ag^1+^Bi^3−^ by the conduction and valence band overlap^17^. SrAgBi is isostructural to CaAuAs where the honeycomb structure features 3 mirror-reflection planes and on each of them opposite mirror eigenvalues intersect and generate nodal loops^18,19^. These loops form a starfruit-like structure in the reciprocal space and cross at higher-symmetry point *A* at the *k*_*z*_ axis. SOC gaps out the nodal loops, creating a pair of type-I Dirac points in the Γ-*A* direction along the the high symmetry *k*_*z*_ axis^19^. Moreover, a type-IV Dirac fermions was predicted in SrAgBi where a band near the type-IV Dirac points is nonlinear, with an additional type-II Dirac point nearby (Fig. 1a)^20^.

In this work we present a combined quantum oscillation, angular resolved photoemission (ARPES) and first-principle calculation study of the electronic states at the Fermi level of SrAgBi. We find evidence for three-dimensional (3D) type-II Dirac semimetal state induced by strong SOC. Though defects may destroy Dirac semimetal and induce other electronic states^21,22^, small amount of Ag vacancies in SrAgBi do not influence Fermi surface characteristics. A single tilted type-II Dirac cone is found along Γ-*A* direction in the Brillouin zone of SrAgBi at the Fermi energy. The SOC-related mechanism of tilted Dirac cone engineering could be used in other charge-balanced materials, paving the way for exotic high-mobility quantum states of interest in optics and spintronics^17,23–30^.

## Results and discussion

### Crystal and band structure

SrAgBi is of the ZrBeSi structure type (Supplementary Note 1), a simple ordered variant of the AlB_2_ structure^16^. The structure can be viewed as flat honeycomb layers of alternating Ag and Bi atoms, both 3-bonded, spaced between slabs of Sr atoms (Fig. 1). This is similar to isostructural KZnBi where K is stuffed in between honeycomb ZnBi layers^31,32^. In SrAgBi Ag-Bi bonds are 2.81 Å, closely matching the sum of the Pauling covalent radii of Ag (1.342 Å) and Bi (1.510 Å), respectively. This is a very strong indication that the Ag-Bi interactions are covalent in nature and that the structure can be rationalized as polyanionic [AgBi]^2−^ layers separated by Sr^2+^ cations.

There are three independent sites in the asymmetric unit (Fig. 1) and based on the refinements, the structure appears to be devoid of disorder on two of them. The nearly spherical shape of the anisotropic displacement parameters for Sr and Bi are a testament to this conjecture (Supplementary Note 1) whereas the extraordinary high value for the *U*_33_ parameter for the Ag atoms has been noted before in the original structure determination^16^, however, it has been attributed to a strong vibration component along the *c*-axis, indicating the tendency of Ag to form an interlayer bond. Since layer to layer separation is over 4.2 Å, such vibration must be considerable. From our interpretation of the diffraction data it appears that the elongation of the anisotropic displacement parameters of the Ag atom is routed in the presence of defects on Ag site where ~1 of 12 atoms are missing as well as in the buckling of the flat layers. The origins of the Ag-defect formation in the Sr-Ag-Bi 1-1-1 phase are presently not understood. However, we must recall that a stoichiometric SrAgBi should be rationalized as Sr^2+^[AgBi]^2−^ (Supplementary Note 2), a valence electron count akin to the Zintl phases and the valence compounds. As such, SrAgBi will be expected to be a small gap semiconductor or a bad metal.

**Fig. 2:**
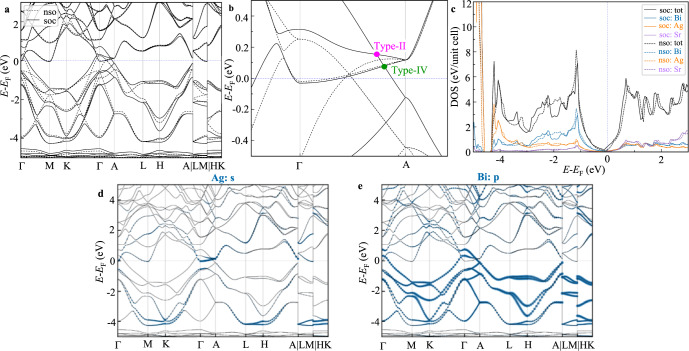
Band structures and density of states (DOS) of SrAgBi. Band structure **a** and details near the Fermi level in Γ-*A* direction **b** with (soc) and without (nso) SOC for SrAgBi . Total and atom-resolved DOS near the Fermi level (*E*_F_) of SrAgBi with and without SOC **c**. Orbital resolved band structure of SrAgBi depicting Ag *s* **d** and Bi *p* **e** orbital contributions.

As shown in Fig. 2a, b, when SOC is included, SrAgBi turns into a Dirac semimetal with one Dirac point locating on the Γ–A line at ~80 meV. From the orbital-resolved density of states (DOS) it is seen that SOC increases DOS in the Fermi energy (*E*_F_) region (Fig. 2c). Near *E*_F_, orbital contribution of all atomic species is similar with relatively steep piles of DOS around *E*_F_ in about 1 eV energy region. From band structure in the presence of SOC (Fig. 2d, e), one can find that the bands near *E*_F_ are mainly contributed by Bi-*p* and Ag-*s* orbitals, which were chosen as the basis for our Wannier based calculations. Bi *p* orbitals mainly contribute to electron whereas hole states are mainly comprised from Ag *s* orbitals near Γ (Fig. 2b, d, e).

### Electrical transport and thermodynamic properties

**Fig. 3:**
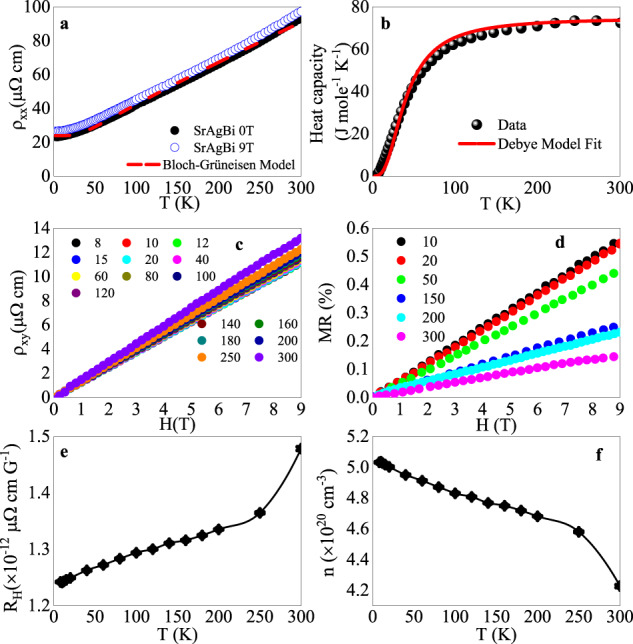
Electronic transport and thermodynamic properties of SrAgBi. **a** Resistivity *ρ*(T) in 0 T and in 9 T with Bloch-Grüneisen fit in the absence of magnetic field. **b** Heat capacity of SrAgBi. Hall *ρ*_*xy*_ **c** and **d** transverse magnetoresistance (MR = $\left(\rho (B)-\rho (0)\right)/\rho (0)\times 100 \%$). Hall coefficient **e** and **f** carrier density.

Electrical resistivity (Fig. 3a) is metallic and shows only a small increase in 9 T magnetic field. In the absence of magnetic field, it is fitted well by the dominant phonon scattering via Bloch-Grüneisen formula:

1 $$\rho (T)={\mu }_{0}+A{\left(\frac{T}{{\theta }_{D}}\right)}^{5}\int\nolimits_{0}^{\frac{{\theta }_{D}}{T}}\frac{{z}^{5}}{({e}^{z}-1)(1-{e}^{-z})}dz$$

where *θ*_*D*_ = 201(6) K is the Debye Temperature. Heat capacity *C*(*T*) is shown in Fig. 3b and it can be only fitted with Debye–Einstein model:

2 $$\begin{array}{lll}{C}_{el+ph}(T)\,=\,\gamma T+(1-\alpha )9nR{\left(\frac{T}{{\theta }_{D}}\right)}^{3}\int\nolimits_{0}^{\frac{{\theta }_{D}}{T}}\frac{{x}^{4}{e}^{x}}{{({e}^{x}-1)}^{2}}dx\\ \qquad\qquad\quad\,\,+\,\alpha 3nR\frac{{({\theta }_{E}/T)}^{2}{e}^{{\theta }_{E}/T}}{{({e}^{{\theta }_{E}/T}-1)}^{2}}\end{array}$$

where *N* = 3 is atomic number and *R* is the universal gas constant. The electronic contribution *γ* = 25(8)mJ/mole ⋅ K^2^. The fit reveals dominant Debye model with Einstein contribution described by parameter *α* = 0.31(3) suggesting the presence of optical phonon modes^33,34^. The model estimates Θ_*D*_ = 206(4) K and Einstein temperature Θ_*E*_ = 50(2) K. Comparison with Θ_*D*_ from Bloch-Grüneisen- resistivity fit implies phonon scattering of *ρ*(*T*) where most acoustical phonon modes take part.

Electronic transport shows a single-band-like behavior, as seen in the linear positive Hall resistivity^35^*ρ*_*xy*_ (Fig. 3c) and unsaturated transverse magnetoresistance MR^36^ (Fig. 3d). Whereas linear magnetoresistance is expected in quantum limit^37,38^ this scenario is unrealistic here since linear MR is observed up to 300 K. Linear positive magnetoresistance at finite temperatures and in low magnetic fields in topologicial band structures is usually considered to arise as a consequence of intra-band or impurity scattering^39–41^. The temperature-dependent Hall coefficient *R*_*H*_ is estimated by the slopes of *ρ*_*xy*_ vs. *H* (Fig. 3e) at different temperatures. Carrier densities *n* are calculated based on the relation ${R}_{H}=\frac{1}{ne}$, where *e* is electronic charge. Carrier density shows reduction with temperature increase with a more pronounced change above 250 K (Fig. 3f). We note that, even though first principle calculations suggest presence of trivial electron pocket at *M* (Fig. 2a), Hall conduction is single-band-like and positive, implying close proximity of Fermi level to the Dirac node. This corresponds to the case when the electron pocket disappears due to energy shift of the Fermi level and the type-II Dirac point dominates^20^.

### Fermi surface characteristics

**Fig. 4:**
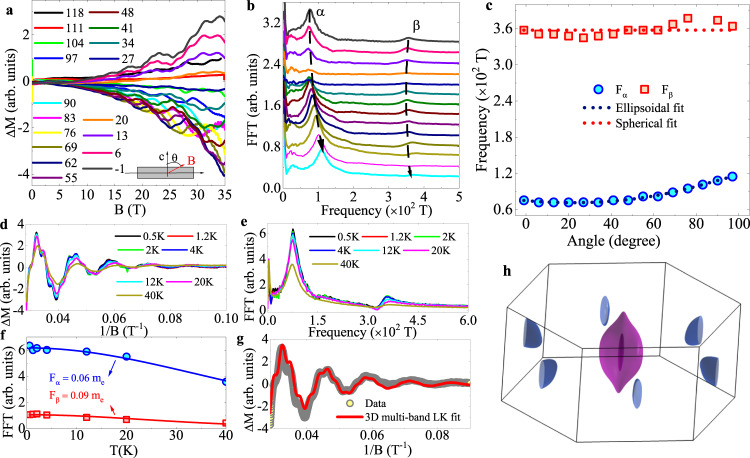
Quantum oscillations and Fermi surface of SrAgBi. **a** Angle-dependent dHvA oscillations; angles between the external magnetic field and crystalline *c*-axis are indicated. **b** Fourier transform amplitude spectrum of Δ*M*, where Δ*M* = *M* − 〈*M*〉, 〈*M*〉 is the smoothed background; offsets are added for clarity. **c** Angular dependence of frequencies *α* and *β*. Ellipsoidal (spherical) model is applied to smaller (larger) frequency *α* (*β*). The good fit demonstrates 3D dimensional character. Temperature dependent dHvA oscillations Δ*M* versus 1/*H.* **d** External magnetic field is fixed along the crystalline *c*-axis. FFT amplitude **e** of Δ*M*, two frequencies *α*, *β* are identified with decreasing amplitudes as temperature is increased. Effective mass on each cyclotron orbit is estimated by temperature factor in Lifshitz-Kosevich (LK) formula. **f** First harmonic-indexed LK formula fit to quantum oscillations. **g** First-principle calculated Fermi surface **h** is contributed by four bands, two Kramers degenerated band pairs, near the Fermi energy *E*_F_.

Angle-dependent de Haas-van Alphen oscillations appear at all angles above 10 T (Fig. 4a). Fast Fourier Transform (FFT) to the dHvA response (Fig. 4b) reveals traces of two dominant frequencies *α*, and *β*, indicating two Fermi surface pockets are detected. Angular evolution of the Fermi surface part associated with frequency *F*_*α*_ = 75.5(4) T is well explained by the ellipsoidal model:

3 $$F(\theta )=\frac{A}{\sqrt{{\epsilon }^{2}{\sin }^{2}(\theta +\phi )+{\cos }^{2}(\theta +\phi )}}$$

where *ϵ* is ellipse eccentricity and *ϕ* is a phase (Fig. 4c). Interestingly, frequency *F*_*β*_ = 358(1) T shows very small angular evolution and can also be fitted with ellipsoidal model for *ϵ* = 0, i.e. a circle. Detected Fermi surface pockets are rather similar to those of Dirac semimetal CaAuAs^42^. The extrema of the Fermi surface cross section areas *S*_*F*_ are estimated from the Onsager relation^43^*F* = (*c*ℏ/2*πe*)*S*_*F*_. For *α* and *β* frequencies we obtain *S*_*F*,*α*_ = 0.720(4) nm^−2^ and *S*_*F*,*β*_ = 3.41(1) nm^−2^. Assuming the circular cross section of Fermi surface *A*_*F*_ = *π*${k}_{F}^{2}$ we obtain *k*_*F*,*α*_ = 0.479(1) Å^−1^ and *k*_*F*,*β*_ = 1.042(1) Å^−1^.

Temperature-dependent dHvA oscillations for magnetic field parallel to *c*-axis are observed above 10 T in all magnetic fields. Bacground-free signal Δ*M* = *M* − 〈*M*〉 where smoothed backgrounds 〈*M*〉 is subtracted from original oscillations *M* is presented in Fig. 4d as a function of inverse field. In the FFT (Fig. 4e) amplitudes of two frequencies *F*_*α*_, *F*_*β*_ are temperature-dependent. Via temperature factors *R*_*T*_ in Lifshitz-Kosevich (LK) formula^43,44^, effective masses associated with both Fermi surface pockets can be estimated from the amplitude of FFT as a function of temperature:

4 $${R}_{T}=\frac{\alpha {m}^{* }T/H}{\sinh (\alpha {m}^{* }T/H)}$$

where *α* = 2*π*^2^*K*_*B*_/*e*ℏ ≈ 14.69 T/K and m^*^ = *m*/*m*_*e*_ is the effective mass of the cyclotron orbit. In our experiment, 1/*H* = (0.1+0.0286)/2, the upper and lower limit of inverse field, thus the obtained masses associated with *F*_*α*_ and *F*_*β*_ are 0.092(9) *m*_*e*_ and 0.060(2) *m*_*e*_, respectively. The very small effective masses in SrAgBi, less than a tenth of free electron mass, are consistent with electronic state in a Dirac semimetal. Using *v*_*F*_ = ℏ*k*_*F*_/m^*^v_*F*,*α*_ = 6.0(6) × 10^5^ m/s and *v*_*F*,*β*_ = 2.01(7) × 10^6^ m/s can be obtained. These are very high Fermi velocities, higher when compared to canonical Dirac semimetals Cd_3_As_2_, Na_3_Bi, ZrTe_5_, black phosphorus or to Weyl semimetals such as WTe_2_ or NbP^45–50^.

Next, we use LK formula^43,44^ with Berry phase to fit oscillatory amplitude at 0.5 K:

5 $${{\Delta }}M\propto\, -\,{H}^{\lambda }{R}_{T}{R}_{D}{R}_{S}sin\left[2\pi \left(\frac{F}{H}-\gamma -\delta \right)\right]$$

where *R*_*T*_ is the temperature factor; *R*_*D*_ = *exp*( − αm^*^T_*D*_/*H*), and *R*_*S*_ = *cos*(πgm^*^/2) are field and spin-damping factors; *α* ≈ 14.69 T/K and m^*^ = *m*/*m*_*e*_. For 3D Fermi surface sheets exponent *λ* is 1/2, *δ* either 1/8 or − 1/8^36^. Since multiple 3D Fermi surface sheets are detected in quantum oscillation experiment, we apply two-band LK formula and we present fit in Fig. 4f. For different *δ*, the fit gives *γ*_*α*_ = 0.031(2), *γ*_*β*_ = − 0.02(1) for *δ* = 1/8, and *γ*_*α*_ = − 0.002(1), *γ*_*β*_ = − 0.013(7) for *δ* = − 1/8. The *γ* is related to Berry phase *ϕ*_*B*_ by $\gamma =\frac{1}{2}-\frac{\phi }{2\pi }$, the calculations give Φ_*α*_ = 0.938(4)*π*, Φ_*β*_ = − 0.96(2)*π* when *δ* = 1/8, and Φ_*α*_ = − 0.996(2)*π*, Φ_*β*_ = − 0.97(1)*π* when *δ* = − 1/8. This confirms non-trivial Berry phases and topological Dirac states associated with both observed Fermi surface pockets.

We estimate Dingle temperature *T*_*D*_ from temperature factor *R*_*D*_ in the LK formula^44^. *T*_*D*_ is associated with scattering rate *τ* of fast moving electrons caused by electronic interactions and defects in the crystal via $\tau =\frac{\hslash }{2\pi {k}_{B}{T}_{D}}$. From the fit, Dingle temperatures for frequencies *α* and *β* are *T*_*Dα*_ = 63(1) K and *T*_*Dβ*_ = 54(6) K for *δ* = − 1/8. Scattering rates are *τ*_*α*_ = 1.93(3) × 10^−14^ s, *τ*_*β*_ = 2.2(3) × 10^−14^ s. Mobility estimate $\mu =\frac{e\tau }{{m}^{* }}$ gives *μ*_*α*_ = 3.7(4) × 10^2^ cm^2^V^−1^s^−1^, *μ*_*β*_ = 6.6(8) × 10^2^ cm^2^V^−1^s^−1^.

Fermi surface of SrAgBi is likely close to compensated with similar electron and hole concentration since Hall resistivity *ρ*_*xy*_ is linear in magnetic fields (Fig. 3c) whereas two different frequencies are detected in quantum oscillation experiment that should correspond to two Fermi surface crossing bands^51–53^. This suggests two pockets at the Fermi surface but also implies that small Ag vacancy defects (Supplementary Note 1) do not have significant impact on near charge compensation and overall Hall conduction^35^. The proposed Dirac Semimetal state for SrAgBi features two Dirac points in Γ-*A* direction, a type-II Dirac state with linear band crossing and a type-IV Dirac point arising from non-linear bands^20^. Both are very close in the Brollouin zone. SrAgBi Fermi surface consists of an electron pocket and a hole pocket of a type-II Dirac point for *E* = 0 eV, but for small positive shift of Fermi level the electron pocket touches the hole pocket and type-IV Dirac point emerges at the touching points^20^. Our first-principles calculations confirm the existence of only one type-II Dirac point in Γ-*A* direction arising from tilted linear crossing bands, induced by strong SOC. There is one electron pocket centered at Γ surrounded by a concentric hole pocket (Fig. 4h). Additionally, there is one electron pocket located at *M* in the Brillouen zone. This is consistent with the presence of electron and hole Fermi surface pockets and with non-trivial character of both pockets observed in quantum oscillations. The smaller (larger) crosssection of *F*_*α*_ (*F*_*β*_) indicates that it comes from the electron (hole) parts of the Fermi surface at Γ whereas *M* pocket is not detected.

**Fig. 5:**
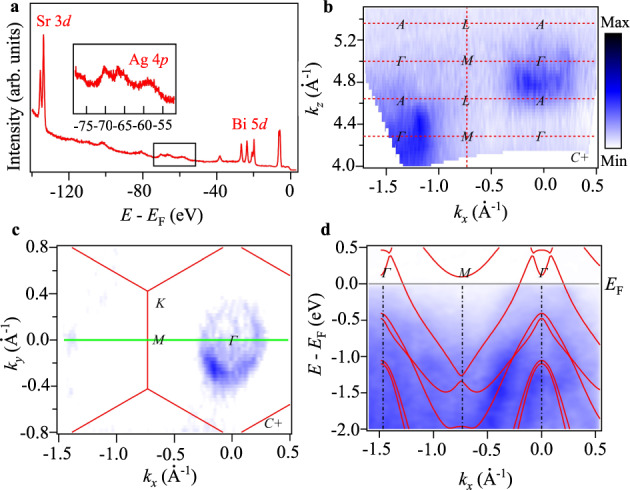
Angular-resolved photoemission spectroscopy of SrAgBi. **a** Core level spectroscopy of SrAgBi, the insert panel shows the zoom-in curves marked by the black box. **b** Fermi Surface map in *A*Γ*ML* plane measured by varying photon energies from 60 eV to 120 eV with circular polarized light. The red dashed lines indicate the BZ boundary and high symmetry lines. **c** ARPES intensity maps at *E*_F_ showing the FS of (001) surface. The boundary of surface BZs are marked by red hexagons. **d** ARPES intensity maps along *G*–*M* high symmetry line (green solid line in **c** with the calculated band structure overlaid). The high symmetry points are marked by dot-dashed lines.

To further investigate the electronic structures of SrAgBi and understand the non-trivial topology within, we conduct angle-resolved photoemission spectroscopy (ARPES) on the (001) surface of SrAgBi, as demonstrated in Fig. 5. The photon energies of incident light in the ARPES experiment are in the ultra-violet range to get the best energy and momentum. Multiple light polarizations are used to probe certain orbital characters. In the core-level spectroscopy performed at the cleaved fresh sample surface (Fig. 5a), all elements in SrAgBi can be identified. We turn to the electronic structures perpendicular to the cleaved (001) surface. The *k*_*z*_ intensity map along Γ–*M* direction (Fig. 5b), which was measured by varying the photon energies from 60 eV to 120 eV, shows clear Fermi pockets around Γ. It indicates the three-dimensional character of SrAgBi, consistent with the angle-dependent dHvA oscillations demonstrated above. It indicates the three-dimensional character of SrAgBi, consistent with the angle-dependent dHvA oscillations demonstrated above. Figure 5c shows the in-plane Fermi surface measured by the photon energy probing the photoelectrons at *k*_*z*_ = 14 *π*/*c* plane. Only one circular hole pocket can be identified at the center of the Brillouin zone (BZ). Combining the in-plane and *k*_*x*_*k*_*z*_ Fermi surface structure, only one hole pocket exists in hole BZ, indicating the hole-doped nature of SrAgBi. In Fig. 5d, we show the electronic structure along Γ-*M* direction with the calculated band structures plotted above. To match the *k*_*F*_ of the hole pocket at Γ, we rigidly shifted the calculations downward around 150 meV. The sufficient consistency between calculations and measured band structures indicates the validation of theory prediction, supporting the existence of type-II Dirac point above the Fermi level.

In conclusion, charge-balanced semimetal SrAgBi hosts type-II Dirac node with small carrier mass and very high Fermi velocity. The *v*_*F*,*β*_ for the hole pocket is among the highest observed in known Dirac semimetals. The compensated Fermi surface of SrAgBi shows insignificant perturbation by Ag vacancy defects whereas the states with non-trivial Berry phase in the Γ-*A* direction dominate electronic transport. This testifies SrAgBi to be a robust material platform for fabrication of nanostructured and thin film-based objects of interest in optoelectronics and for observation of exotic quantum states.

## Methods

### Single crystal growth and characterization

Raw materials, Sr,Ag and Bi were mixed in a ratio of 1:1:4 in an alumina crucible and sealed in a quartz tube, which was then Argon-flushed and sealed in vacuum. The ampoule was heated to 900^∘^C and kept at that temperature for 24 h. Crystals were decanted at 500^∘^C in a centrifuge after 10 days of cooling. Light grey shiny crystals in a typical size of 1 mm × 1 mm × 1 mm were obtained. Excess residual flux was cleaned by polishing before measurements. The average stoichiometry was determined by examination of multiple points on polished crystal surfaces using energy-dispersive X-ray spectroscopy (EDS) in a JEOL LSM-6500 scanning electron microscope. The average atomic percent ratio Sr:Ag:Bi was 31(3):34(2):34(2), consistent with stoichiometry close to expected SrAgBi. Resistivity was measured by conventional four-wire method in a Quantum Design PPPMS-9. Small cuboid specimen were taken to National High Magnetic Field Laboratory Tallahassee for measurements of temperature- and angular-dependent de Haas-van Alphen (dHvA) oscillations, where the field ranged from 0 to 35 Tesla. The crystal was mounted on the rotator; the angle between magnetic field and crystalline *c*-axis was indicated.

Single-crystal X-ray diffraction intensity data sets were collected for a crystal cut under a microscope to dimensions 0.08 ⋅ 0.06 ⋅ 0.05 mm^3^. The crystal was mounted on a low background plastic holder using Paratone N oil, transferred to the goniometer, and placed under the cold stream of nitrogen gas. Data acquisition took place at 200 K on a Bruker SMART APEX CCD diffractometer using graphite monochromatized Mo K*α* radiation (*λ* = 0.71073 *Å*). Intensity data were collected in 2 batch runs at different *ω* and *φ* angles with an exposure time of 6 sec/frame. A total of 1378 reflections (2*θ*_*max*_ ~ 60^∘^, 92% coverage) were collected, 124 of which were symmetry-unique (T_*min*_/T_*max*_ = 0.07/0.13; R_*int*_ = 0.066). The data collection, data reduction and integration, as well as refinement of the cell parameters were carried out using the Bruker-provided programs (SAINT; Bruker AXS Inc., Madison, Wisconsin, USA, 2014). Semi-empirical absorption correction was applied with the aid of the SADABS software package (SADABS; Bruker AXS Inc., Madison, Wisconsin, USA, 2014). The structure was subsequently solved by direct methods and refined on *F*^2^ (9 parameters) with the aid of the SHELXL package^54^. All atoms were refined with anisotropic displacement parameters with scattering factors (neutral atoms) and absorption coefficients^55^. We note that the [AgBi] layers could be slightly puckered, as evidenced by the elongated thermal parameter, therefore non-centrosymmetric structure in *P6*_*3*_*mc* might be considered in a forthcoming more detailed crystallographic study. The corresponding crystallographic information file (CIF) has been deposited with the Cambridge Crystallographic Database Centre (CCDC) and can be obtained free of charge via http://www.ccdc.cam.ac.uk/conts/retrieving.html (or from the CCDC, 12 Union Road, Cambridge CB2 1EZ, UK; Fax: +44-1223-336033; E-mail: deposit@ccdc.cam.ac.uk)—depository number xxxxxx.

### First-principles calculations

We performed the first-principles calculations based on the density functional theory (DFT) using projector augmented wave (PAW) method^56,57^ implemented in the Vienna ab initio simulation package (VASP)^58,59^ to obtain the electronic structures. The generalized gradient approximation (GGA) with exchange-correlation functional of Perdew, Burke and Ernzerhof (PBE) for the exchange-correlation functional^60^ was adopted. The kinetic energy cutoff was set to 300 eV for the plane wave bases. The BZ was sampled by Γ-centered Monkhorst-Pack method^61^ with a 9 × 9 × 6 -mesh for the 3D periodic boundary conditions in the self-consistent process. The Wannier-based tight-binding (TB) model under bases of the Sr-*d*, Ag-*s*, and Bi-*p* orbitals (Fig. 2) is extracted from the DFT calculations^62^ for the calculations of Fermi surface^63^.

### Angle-resolved photoemission spectroscopy

ARPES and X-ray photoemission (XPS) data presented in main text were measured at the ULTRA endstation at the Surface/Interface Spectroscopy (SIS) beamline of the Swiss Light Source. The data were acquired with a Scienta Omicron DA30L hemispherical analyzer. The energy and angular resolution are better than 20 meV and 0.1^∘^. The measurements were performed at a temperature of 12 K in a base pressure better than 5 × 10^−10^ Torr.

### X-ray photoemission spectroscopy

For accurate valence determination XPS experiments were also carried out in an ultrahigh vacuum (UHV) system with base pressures of 5 × 10^−9^ Torr and equipped with a hemispherical electron energy analyzer (SPECS, PHOIBOS 100) and twin anode X-ray source (SPECS, XR50). Al K_*α*_ (1486.7 eV) radiation with power of 300 W(15 kV, 20 mA) was used for photoemission excitation. The angle between the analyzer and X-ray source is 45^∘^ and photoelectrons were collected along the sample surface normal direction. In order to remove potential surface contaminations and oxidized layers, sample was bombarded in situ by Ar^+^ ions accelerated to 2 keV under pressure of 2 × 10^−5^ Torr of Ar gas for 60 min. XPS data was analyzed using Casa XPS and peak positions were calibrated using residual adventitious carbon C 1s at 284.8 eV.

### Supplementary information

Supplement

## Data Availability

The data that support the findings of this study are available from the corresponding authors upon reasonable request.
