# Supplementary material for: Robust three-dimensional type-II Dirac semimetal state in SrAgBi
Source: NPJ Quantum Mater. 2023 May 6;8(1):20. doi: 10.1038/s41535-023-00549-8 (PMC11041807; doi:10.1038/s41535-023-00549-8)
Supplement: Supplementary file 1 — Supplement [file 41535_2023_549_MOESM1_ESM.pdf]

Supplement for: Robust three-dimensional type-II Dirac semimetal state in SrAgBi

Zhixiang Hu,[1,2] Junze Deng,[3,4] Hang Li,[5] Michael Ogunbunmi,[6] Xiao Tong,[7] Qi Wang,[8,§] David Graf,[9] Wojciech Radoslaw Pudełko,[5,10] Yu Liu,[1,‖] Hechang Lei,[8] Svilen Bobev,[6] Milan Radovic,[5] Zhijun Wang[3,4] and Cedomir Petrovic[1,2]

[1] *Condensed Matter Physics and Materials Science Department, Brookhaven National Laboratory, Upton, New York 11973, USA*

[2] *Department of Material Science and Chemical Engineering, Stony Brook University, Stony Brook, New York 11790, USA*

[3] *Beijing National Laboratory for Condensed Matter Physics and Institute of Physics, Chinese Academy of Sciences, Beijing 100190, China*

[4] *University of Chinese Academy of Sciences, Beijing 100049, China*

[5] *Swiss Light Source, Paul Scherrer Institut, CH-5232 Villigen, Switzerland*

[6] *Department of Chemistry and Biochemistry, University of Delaware, Newark, Delaware 19716, USA*

[7] *Center of Functional Nanomaterials, Brookhaven National Laboratory, Upton, New York 11973, USA*

[8] *Department of Physics and Beijing Key Laboratory of Opto-electronic Functional Materials Micro-nano Devices, Renmin University of China, Beijing 100872, China*

[9] *National High Magnetic Field Laboratory, Florida State University, Tallahassee, Florida 32306-4005, USA*

[9] *Physik-Institut, Universität Zürich, Winterthurerstrasse 190, CH-8057 Zürich, Switzerland*

(Dated: March 22, 2023)

SUPPLEMENTARY NOTE 1: CRYSTAL STRUCTURE DETAILS

The final Fourier map in the single crystal X-ray diffraction experiment is featureless with the highest residual density and deepest hole of about 1.1 $e^-/Å^3$ and -2.8 $e^-/Å^3$, respectively, both located about 1 Å away from Bi. Occupancies of all atomic sites were checked by refinement of the respective site occupation factors (SOFs). The SOFs or Sr and Bi did not deviate from unity within more than 3 standard deviations, however the SOF for Ag was refined as 0.929(12). Since there was no evidence from energy-dispersive X-ray spectroscopy for the incorporation of lighter metallic elements in the studied specimen, and since this behavior was reproducible for several crystal from different batches, we attribute the SOF deviation from 100% at the Ag site as in indicator that there is a small amount of vacancy defects at this position. This is coupled with the elongated anisotropic displacement parameter discussed later on. Further details of the data collection and structure refinements are given in Table I, the positional and equivalent displacement parameters are listed in Table II, respectively. The final refined structure (ZrBeSi type, Pearson index hP6) is shown in Figure 1 with anisotropic displacement parameters drawn at the 90% probability

Supplementary Table I. Selected single-crystal data collection and structure refinement parameters for $SrAg_{0.93(2)}Bi$ measured at 200 K using $\lambda = 0.71073$ Å. The corresponding crystallographic information file (CIF) has been deposited with the Cambridge Crystallographic Database Centre (CCDC) - depository number - 2208657

Refined composition	$SrAg_{0.93(1)}Bi$
Formula Mass (g/mol)	396.92
Space Group	$P6_3/mmc$ (No. 194)
Z	2
a (Å)	4.8682(6)
c (Å)	8.4621(17)
V (Å^3)	173.68(5)
Density (g/cm^3)	7.59
$\mu_{MoK\alpha}$ (cm^1)	707.4
$R1$ $[I > 2\sigma(I))]^a$	0.0283
$wR2$ $[I > 2\sigma(I))]^a$	0.0582
$\Delta\rho_{max,min}$/e·Å^{-3}	1.10,-2.83

^{a} $R1 = \Sigma|F_0| - |F_c|/\Sigma|F_0|$,

$wR2 = [\Sigma(|F_0^2 - |F_c^2|)^2/\Sigma(wF_0^2)^2]^{1/2}$

w=1/[$\sigma^2F_0^2$+(0.0202·P)^2+(3.032·P)] and P=(F_0^2+2F_c^2)/3

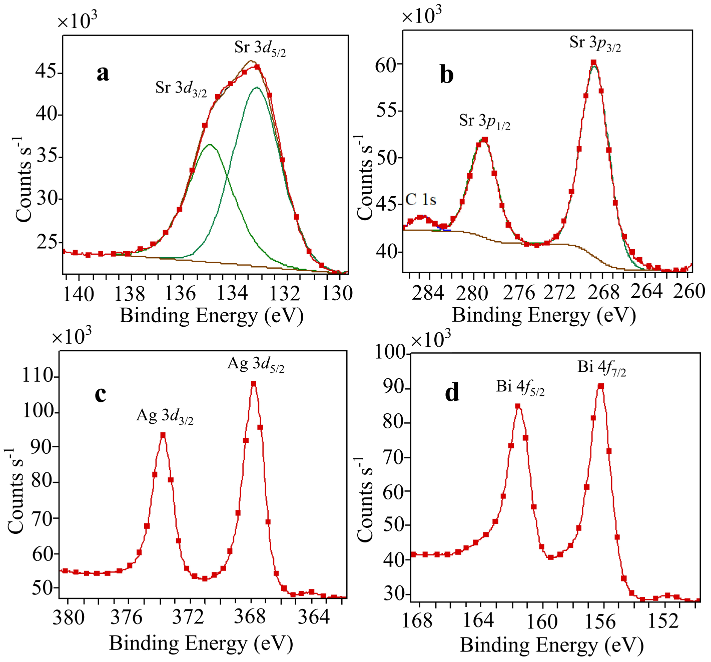

Supplementary Figure 1. XPS spectra for core levels of **a** Sr $3d$, **b** Sr $3p$, **c** Ag $3d$ and **d** Bi $4f$ in SrAgBi crystal.

level.

Supplementary Table II. Atomic coordinates, equivalent Å^2 and anisotropic displacement parameters(Å^2 for SrAg$_{0.93(1)}$Bi at 200 K. U$_{eq}$ is defined as one-third of the trace of the orthogonalized U_{ii} tensor.

Atom	Site	x	y	z	$U_{eq}{}^a$	U_{11}	U_{22}	U_{33}	U_{23}	U_{13}	U$_{12}$
Sr	2a	0	0	0	0.016(1)	0.018(1)	0.018(1)	0.013(1)	0	0	0.009(1)
Ag^b	2c	1/3	2/3	1/4	0.045(1)	0.012(1)	0.012(1)	0.109(1)	0	0	0.006(1)
Bi	2d	1/3	2/3	3/4	0.013(1)	0.010(1)	0.010(1)	0.019(1)	0	0	0.005(1)

Atomic displacement parameters provide information about thermal motion of atomic species; for example U_{33} describes the motion along the crystallographic c-axis.

SUPPLEMENTARY NOTE 2: LABORATORY X-RAY PHOTOEMISSION SPECTROSCOPY

XPS spectra of Sr $3d$ level with deconvoluted spin-orbit doublet of $3d_{5/2}$ and $3d_{3/2}$ components with energy separation of 1.79 eV are shown in Fig. S1a at binding energy of 133.1 eV and 134.9 eV respectively. Clearly, they are the finger prints of the divalent cation of Sr^{2+} [1, 2]. The Sr^{2+} state is further confirmed by Sr $3p_{3/2}$ and Sr $3p_{1/2}$ with binding splitting 10.4 eV at binding energy of 269.0 eV and 279.2 eV, respectively as shown in in Fig. S1b,which are in agreement with that of reported Sr $3p$ for Sr^{2+} [3, 4].

Fig. S1c shows the XPS spectra of the Ag $3d$ with spin-orbit doublet of Ag $3d_{5/2}$ and Ag $3d_{3/2}$ at binding energy of 367.8 eV and 373. 8 eV are associated Ag^{1+} states [5].

Fig. S1d shows XPS spectra of Bi $4f$ level with spin‘orbit doublet of Bi $4f_{7/2}$ and Bi $4f_{5/2}$ at binding energy of 156.2 eV and 161.5 eV , respectively, which shifted to the lower binding energy about -0.8 eV when compared to Bi $4f$ in metallic state. This observation suggests that the Bi is in electronegative anionic state in the compounds. Whereas similar bonding scheme with electronegative bismuth was proposed for another well known topological Dirac semimetal Na$_3$Bi [6], to our best knowledge this is the first experimental observation in a crystal. Bi should be in state of trivalent anion Bi^{3-} in the compound in order to satisfy the compound electroneutrality of Sr^{2+}Ag^{1+}Bi^{3-}. The electronegativity increase in the order of Sr(0.92) $\longrightarrow$ Ag(1.93) $\longrightarrow$ Bi(2.02) support our observation and conclusion.

¶Present address: School of Physical Science and Technology, Shanghai Tech University, Shanghai 201210, China.
||Present address: Los Alamos National Laboratory, Los Alamos, NM 87545, USA.

---

[1] Sharma, N., Shaju, K. M., Subba Rao, V., Chowdari B. V. R., Anodic behaviour and X-ray photoelectron spectroscopy of ternary tin oxides. *J. Power. Sources* **139**, 250-260 (2005).

[2] Misra, S., Gnanasekar, K. I., Subba Rao, R. V. Electrical conductivity and oxygen sensing behavior of $SrSn_{1-x}Fe_xO_{3-\delta}$ (x = 0 - 0.2). *J. Alloys Comp.* **506**, 285-292 (2010).

[3] Kurmaev, E. Z., Glakhov, V. R., Fedorenko, V. V., Elokhina, L. V., Bartkowski, S., Neumann, M., Greaves, C., Edwards, P. P., Al-Mamouri, M., Novikov, D. L. X-ray emission, photoelectron spectra and electronic structure of $Sr_2CuO_2F_{2+\delta}$. *Phys. Rev. B* **52**, 2390 (1995).

[4] Atuchin, V. V., Kesler, V. G., Zaitsev, A. I., Molokeev, M. S., Aleksandrovsky, A. S., Kuzubov, A. A., Ignatova, N. Y. Electronic structure of α-SrB_4O_7: experiment and theory. *J. Phys. Cond. Matt.* **25**, 085503 (2013).

[5] Korduban, O. M., Kryshchuk, T. V., Kandyba, V. O., Trachevskii, V. V. XPS studies of the surface of TiO_2:Ag nanopowders. *Chemistry, Physics and Technology of Surface* **11**, 547-555 (2020).

[6] Dong, X.-X., Chen, J.-X., Wang, Y., Lv, Z.-L., Wang, H.-Y. Electronic, elastic and lattice dynamic properties of the topological Dirac semimetal Na_3Bi. *Materials Research Express* **6**, 076308 (2019).
